# Functional hemispheric disconnection procedures for chronic epilepsy: history, indications, techniques, complications and current practice in Europe. A consensus statement on behalf of the EANS functional neurosurgery section

**DOI:** 10.1016/j.bas.2024.102754

**Published:** 2024-01-28

**Authors:** Olaf E.M.G. Schijns, Daniel Delev, Marec von Lehe, Dirk van Roost, Karl Rössler, Tom Theys, Christian Auer, Thomas Blauwblomme, Marcelo Budke, Alexandre Rainha Campos, Santiago Candela Canto, Hans Clusmann, Christian Dorfer, Georg Dorfmüller, Arild Egge, Lorand Eröss, Sarah Ferrand-Sorbets, Flavio Giordano, Jürgen Honegger, Cihan Isler, Jugoslav Ivanovic, Thilo Kalbhenn, Atte Karppinen, Niklaus Krayenbühl, Rick H.G.J. van Lanen, Carlo E. Marras, Ioannis Mavridis, Daniel Nilsson, Julia Onken, Christian Raftopoulos, Jonathan Roth, Jordi Rumia, Thomas Sauvigny, Didier Scavarda, Karl Schaller, Christian Scheiwe, Sophie Schuind, Alexandra Seromenho-Santos, Kostas Fountas

**Affiliations:** Department of Neurosurgery, Maastricht University Medical Center, Maastricht, the Netherlands; Academic Center for Epileptology, Maastricht University Medical Center and Kempenhaeghe, Maastricht-Heeze, the Netherlands; School for Mental Health and Neuroscience (MHeNs), University Maastricht (UM), Maastricht, the Netherlands; Department of Neurosurgery, RWTH Aachen University Hospital, Aachen, Germany; Department of Neurosurgery, Ruppiner Kliniken, Neuruppin, Germany; Department of Neurosurgery, University Hospital Ghent, Ghent, Belgium; Department of Neurosurgery, Medical University of Vienna, Vienna, Austria; Department of Neurosurgery, University Hospital Leuven, Leuven, Belgium; Department of Neurosurgery, Johannes Kepler University Linz, Kepler University Hospital, Linz, Austria; Department of Pediatric Neurosurgery, Hôpital Necker, Université de Paris, Paris, France; Department of Neurosurgery, Hospital Infantil Universitario Niño Jesús, Madrid, Spain; Department of Neurosurgery, Hospital Santa Maria, Lisbon, Portugal; Department of Neurosurgery, Hospital Sant Joan de Deu, Barcelona, Spain; Department of Neurosurgery, RWTH Aachen University Hospital, Aachen, Germany; Department of Neurosurgery, Medical University of Vienna, Vienna, Austria; Department of Neurosurgery, Hopital Fondation Adolphe de Rothschild, Paris, France; Department of Neurosurgery, Oslo University Hospital, Oslo, Norway; Department of Neurosurgery, National Institute of Mental Health, Neurology and Neurosurgery, Budapest, Hungary; Department of Neurosurgery, Hopital Fondation Adolphe de Rothschild, Paris, France; Department of Neurosurgery, Meyer Children's Hospital IRCCS, University of Florence, Italy; Department of Neurosurgery, University Hospital, Tübingen, Germany; Department of Neurosurgery, Istanbul University-Cerrahpasa, Istanbul, Turkey; Department of Neurosurgery, Oslo University Hospital, Oslo, Norway; Department of Neurosurgery, Bielefeld University, Medical School, Bielefeld, Germany; Department of Neurosurgery, University of Helsinki, Helsinki University hospital, Helsinki, Finland; Department of Neurosurgery, Universitäts-Kinderspital Zürich-Eleonorenstiftung, Zürich, Switzerland; Department of Neurosurgery, Maastricht University Medical Center, Maastricht, the Netherlands; School for Mental Health and Neuroscience (MHeNs), University Maastricht (UM), Maastricht, the Netherlands; Department of Neurosurgery, Ospedale Pediatrico Bambino Gesu, Roma, Italy; Department of Neurosurgery, University General Hospital of Alexandroupolis, Alexandroupolis, Greece; Department of Neurosurgery, Sahlgrenska University hospital, Göteborg, Sweden; Department of Neurosurgery, Charite University hospital, Berlin, Germany; Department of Neurosurgery, Clinique Universitaires Saint Luc, Brussels, Belgium; Department of Neurosurgery, Tel Aviv Sourasky medical center, Tel Aviv, Israel; Department of Neurosurgery, Hospital Sant Joan de Deu, Barcelona, Spain; Department of Neurosurgery, University Medical Center Hamburg-Eppendorf, Hamburg, Germany; Department of Neurosurgery, Hopital La Timone Enfants, Marseille, France; Department of Neurosurgery, Geneva University Medical Center, Geneva, Switzerland; Department of Neurosurgery, University hospital, Freiburg, Germany; Department of Neurosurgery, Hopital Erasme, Brussels, Belgium; Department of Neurosurgery, Centro Hospitalar de Lisboa Ocidental, Lisbon, Portugal; Department of Neurosurgery, University of Thessaly Medical School, Larissa, Greece

**Keywords:** Epilepsy surgery, Functional hemispherotomy, Hemispheric disconnection, Indications, Techniques, Complications

## Abstract

**Introduction:**

The surgical procedure for severe, drug-resistant, unilateral hemispheric epilepsy is challenging. Over the last decades the surgical landscape for hemispheric disconnection procedures changed from anatomical hemispherectomy to functional hemispherotomy with a reduction of complications and stable good seizure outcome. Here, a task force of European epilepsy surgeons prepared, on behalf of the EANS Section for Functional Neurosurgery, a consensus statement on different aspects of the hemispheric disconnection procedure.

**Research question:**

To determine history, indication, timing, techniques, complications and current practice in Europe for hemispheric disconnection procedures in drug-resistant epilepsy.

**Material and methods:**

Relevant literature on the topic was collected by a literature search based on the PRISMA 2020 guidelines.

**Results:**

A comprehensive overview on the historical development of hemispheric disconnection procedures for epilepsy is presented, while discussing indications, timing, surgical techniques and complications. Current practice for this procedure in European epilepsy surgery centers is provided. At present, our knowledge of long-term seizure outcomes primarily stems from open surgical disconnection procedures. Although minimal invasive surgical techniques in epilepsy are rapidly developing and reported in case reports or small case series, long-term seizure outcome remain uncertain and needs to be reported.

**Discussion and conclusion:**

This is the first paper presenting a European consensus statement regarding history, indications, techniques and complications of hemispheric disconnection procedures for different causes of chronic, drug-resistant epilepsy. Furthermore, it serves as the pioneering document to report a comprehensive overview of the current surgical practices regarding this type of surgery employed in renowned epilepsy surgery centers across Europe.

## 1 Introduction

Hemispheric diseases, like perinatal stroke, Rasmussen encephalitis, hemimegalencephaly or Sturge-Weber syndrome, can cause a severe, drug-resistant epilepsy (DRE), especially in pediatric patients, frequently leading to an increased morbidity and mortality (Varadkar et al., 2014; Di Rocco and Tamburrini, 2006). Hemispheric disconnection procedures (HDP) offer a well-established curative epilepsy treatment leading to seizure freedom in a high percentage of patients (Kim et al., 2018; Hale et al., 2023). Over the decades of the 20th century these surgical procedures changed from anatomical disconnection with resection of large parts of the brain to functional disconnection and minimal resection of brain tissue (Schramm, 2002). This consequently lowered the perioperative complication risk, without influencing the good seizure outcome percentage (Schramm et al., 1995a). These functional disconnection techniques consist of two ‘main’ techniques, the vertical and the lateral hemispheric disconnection, with some variants (Schramm, 2002; Schramm et al., 1995a; Fallah et al., 2021). Very recently minimal invasive surgical techniques have appeared for this indication, like the magnetic resonance imaging (MRI)-guided laser interstitial thermal therapy, and radiofrequency ablation but currently long-term follow-up data are not yet available, so the definitive post-treatment seizure outcome cannot be compared with that of open surgical procedures (Badger et al., 2020; Curry et al., 2012; Sarat Chandra et al., 2021a). Here, members of the European Association of Neurosurgical Societies (EANS) functional neurosurgery section and invited European renowned experts in the field of epilepsy surgery provide a consensus statement on the history, indication, timing, techniques, complications and current European practice for hemispheric disconnection procedures in drug-resistant epilepsy.

## 2 Methods

We aimed to substantiate this consensus statement by including and referring to literature on the aforementioned topics of hemispheric disconnection procedures in drug-resistant epilepsy. Therefore, available literature was searched using the search engines PubMed, Embase, and the Cochrane Library. For this review the Preferred Reporting Items for Systematic Reviews and Meta-Analyses (PRISMA) guidelines and extension for Scoping Reviews were applied where applicable (Page et al., 2021; Tricco et al., 2018). Selection of search terms was based on an explorative literature search with the following search terms alone or in combination: hemispherectomy, hemispheric surgery, hemispherotomy, hemidecortication, hemicorticectomy, (drug-resistant or intractable) epilepsy, and seizure, along with search terms for the individual topics: history, indication, timing, surgical technique, complications and practice. The search was last performed on February 25, 2023. The bibliographies of original articles and reviews were also searched for additional relevant publications. Searches were not restricted by publication date. The search yielded a total of 626 articles.

Titles and abstracts were screened and used in this review based on their applicability to one of the chapters/sections: 1) history; 2) indications; 3) timing; 4) techniques; 5) complications; 6) current practice. The inclusion criteria were peer-reviewed research articles or reviews, retrospective or prospective studies describing hemispheric disconnection procedures in drug-resistant epilepsy; pediatric and adult patients. Exclusion criteria were non-epileptic indications; article focus on non-disconnection procedures; article in non-European language. Editorials, notes, letters and single case reports were also excluded. Articles deemed applicable to this review had their full-text copies acquired.

A PICO question (P: Patient/Problem, I: Intervention, C: Comparison, O: Outcome) was formulated to lead the selection process: the population was defined as patients (both pediatric and adults) with drug-resistant epilepsy, the intervention and comparison was any hemispheric disconnection procedure, and outcomes included surgical indications (acquired pathologies; malformations of cortical development; progressive pathologies), timing, techniques (anatomical disconnection; functional disconnection; new developments) and complications (overview and avoidance).

A task force composed of members of the EANS functional neurosurgery section and invited European renowned experts in the field of epilepsy surgery was created to articulate this consensus paper on the history, indication, timing, techniques, complications and current European practice for hemispheric disconnection procedures in drug-resistant epilepsy, relying on the available literature. Furthermore, a questionnaire on the current practice of hemispheric disconnection procedures in their centers (Supplement 1) was sent to all co-authors. The answers are summarized in Table 1. Consensus was elaborated after review of the literature and discussion among the experts.

**Table 1:** Overview of European hospitals performing hemispheric disconnective procedures for epilepsy.

Country/HDP center	Start HDP surgery	Nr. of centers	Nr. of surgeons	Nr. of annual HDP	Type HDP	Subtype HDP	Difference in complications lateral vs vertical	Approach	Indications	Age group	Frequency HDP surgery last 5–10 years
**Germany**		5									
Center 1	2010		1	5	Functional hemispherotomy	Lateral/transsylvian	n/a	open	Hemimegalencephaly Rasmussen encephalitis Perinatal infarction Sturge-Weber syndrome HHE syndrome	adult + pediatric	no change
Center 2	2000		1	3	Functional hemispherectomy	Lateral/transsylvian	n/a	open + endoscopic	Hemimegalencephaly Rasmussen encephalitis	pediatric	increase
Center 3	1980		1	18	Functional hemispherotomy	Lateral/transsylvian	Yes, less hydrocephalus in case less tissue is removed	open	Hemimegalencephaly (17 %) Rasmussen encephalitis (16 %) Perinatal infarction (39.6 %) Sturge-Weber syndrome (2.6 %) Other (24.8 %)	adult (13 %) pediatric (87 %)	increase
Center 4	1998		2	5–10	Functional hemispherotomy + Functional hemispherectomy (hemimegalencephaly)	Lateral/transsylvian Vertical parasagittal hemispherotomy	no	Open + endoscopic	Hemimegalencephaly Rasmussen encephalitis Perinatal infarction Sturge-Weber syndrome HHE syndrome	adult + pediatric	no change
Center 5	No report										
**The Netherlands**		2									
Center 1	2012		2	1–2	Functional hemispherotomy	Lateral/transsylvian	n/a	open	Hemimegalencephaly Rasmussen encephalitis Perinatal infarction Sturge-Weber syndrome HHE syndrome	adult + pediatric	decrease
Center 2	No report										
**Belgium**		3									
Center 1	2000		1	2–6	Functional hemispherectomy	Lateral/transsylvian Vertical parasagittal hemispherotomy (2002–2012)	no	open	Hemimegalencephaly Rasmussen encephalitis Perinatal infarction Sturge-Weber syndrome HHE syndrome	adults + pediatric	slight increase
Center 2	2000		1	0–2	Functional hemispherotomy	Lateral/transsylvian	n/a	open	Hemimegalencephaly Rasmussen encephalitis Perinatal infarction Sturge-Weber syndrome HHE syndrome	pediatric	No change
Center 3	2000		1	20	Functional hemispherotomy	Modified vertical parasagittal hemispherotomy + Lateral/transsylvian	MVPH is more rapid and associated with less blood loss	open	Hemimegalencephaly Rasmussen encephalitis Perinatal infarction Sturge-Weber syndrome HHE syndrome	pediatric	decrease
**Switzerland**		2									
Center 1	2000		2	2–3	Functional hemispherectomy	Lateral/transsylvian	n/a	open	Hemimegalencephaly Rasmussen encephalitis Perinatal infarction Sturge-Weber syndrome HHE syndrome	adult + pediatric	no change
Center 2	1950 hemispherectomy 2010 hemispherotomy		1	2–3	Functional hemispherotomy	Lateral/transsylvian	n/a	open	Hemimegalencephaly Rasmussen encephalitis Perinatal infarction Sturge-Weber syndrome other: extended focal cortical dysplasia	adult + pediatric	increase
**Austria**		2									
Center 1	1990		2	3–5	Functional hemispherotomy	Vertical parasagittal hemispherotomy	n/a	open	Hemimegalencephaly Rasmussen encephalitis Perinatal infarction Sturge-Weber syndrome HHE syndrome	pediatric	decrease
Center 2	2010		1	1–2	Functional hemispherotomy	Vertical parasagittal hemispherotomy	n/a	open	Hemimegalencephaly Perinatal infarction Sturge-Weber syndrome	pediatric	increase
**France**		3									
Center 1	1980		1	3–10	Functional hemispherotomy	Vertical, Midline interhemispheric sagittal hemispherotomy	n/a	open	Hemimegalencephaly (32 %) Rasmussen encephalitis (23 %) Perinatal infarction (23 %) Sturge-Weber syndrome (7 %) HHE syndrome (0 %) Other. Multilobar FCD (13 %)	pediatric	increase
Center 2	1990		2	10–15	Functional hemispherotomy	Vertical parasagittal hemispherotomy	n/a	open	Hemimegalencephaly Rasmussen encephalitis Perinatal infarction Sturge-Weber syndrome HHE syndrome Other: Sub-/hemispheric cortical dysplasia, other than Hemimegalencephaly Polymicrogyria with hemispheric epilepsy A few patients with tuberous sclerosis complex, but associated either with HME or other hemispheric dysplasia	adult + pediatric	no change
Center 3	2000–2010		1	3–4	Functional hemispherotomy	Vertical parasagittal hemispherotomy	duration	open	Hemimegalencephaly Rasmussen encephalitis Perinatal infarction Sturge-Weber syndrome	pediatric	increase
**Spain**		3									
Center 1	1980		1	8–10	Functional hemispherotomy	Lateral/transsylvian	n/a	open	Hemimegalencephaly Rasmussen encephalitis Perinatal infarction Sturge-Weber syndrome Other: Multilobar cortical dysplasia	pediatric	increase
Center 2 + 3	1998		2	3–5	Functional hemispherotomy + functional hemispherectomy (hemimegencephaly)	Lateral/transsylvian + vertical parasagittal hemispherotomy (in TSC)	no	open + MRIgLITT	Hemimegalencephaly/Extensive hemispheric malformation (dysplasia) Rasmussen encephalitis Perinatal infarction (poroencephalic cyst) Sturge-Weber syndrome	adult + pediatric	increase
Portugal		3									
Center 1	2005		1	1	Functional hemispherotomy	Lateral/transsylvian	no	open	Hemimegalencephaly Rasmussen encephalitis Perinatal infarction Sturge-Weber syndrome HHE syndrome Other – Tuberous sclerosis (Posterior Quadrantic Disconnection)	adult + pediatric	increase
Center 2	2000–2010		1 + 1 in training	1	Functional hemispherotomy	Lateral/transsylvian	no	open	Hemimegalencephaly Rasmussen encephalitis Perinatal infarction Sturge-Weber syndrome Other: Multilobar cortical dysplasia	pediatric	no change
**Italy**		5									
Center 1	2000		1	5	Functional hemispherotomy + functional hemispherectomy	Vertical parasagittal hemispherotomy Modified vertical parasagittal hemispherotomy	no	open	Hemimegalencephaly Rasmussen encephalitis Perinatal infarction Sturge-Weber syndrome HHE syndrome Other any other hemispheric lesions (i.e., head injury, infection)	pediatric	increase
Center 2	2010		1	?	Functional hemispherotomy	Vertical parasagittal hemispherotomy	A smaller higher rate of hydrocephalus in the vertical approach (Delalande) compared to the lateral (Villemure)	open	Hemimegalencephaly Rasmussen encephalitis Perinatal infarction	pediatric	increase
**Norway**		1									
Center 1	2011		1	1–3	Functional hemispherotomy	Lateral/transsylvian	n/a	open	Hemimegalencephaly Rasmussen encephalitis Perinatal infarction	pediatric	increase
**Sweden**		1									
Center 1	1990		1	1–3	Functional hemispherotomy	Lateral/transsylvian (1995–2010) Vertical parasagittal hemispherotomy (2011-now)	shorter operating time, less blood loss, better outcome for vertical approach	open	Hemimegalencephaly Rasmussen encephalitis Perinatal infarction Sturge-Weber syndrome HHE syndrome	adult + pediatric	increase
**Finland**		2 (1 for HDP)									
Center 1	1995		1	2	Functional hemispherotomy	Vertical parasagittal hemispherotomy	n/a	open	Hemimegalencephaly Rasmussen encephalitis Perinatal infarction Surge-Weber Syndrome Other: Polymicrogyria Hemispheric dysgenesis Posttraumatic	adult + pediatric	no change
**Greece**											
Center 1	2022	1	1	1	Functional hemispherotomy	Lateral/transsylvian	n/a	open	Rasmussen encephalitis Sturge-Weber syndrome	adult	no change
**Israël**	1990–2000	3	1	5–10	Functional hemispherotomy	Vertical parasagittal hemispherotomy + peri-insular hemispherotomy	no	open	Hemimegalencephaly Rasmussen encephalitis Perinatal infarction Sturge-Weber syndrome HHE syndrome	adult + pediatric	Increase
**Bosnia Herzegovina**											
Center 1	2010	1	1	1–2	Functional hemispherotomy	Lateral/transsylvian	n/a	open	Rasmussen encephalitis	pediatric	no change
**Turkey**											
Center 1	2000	3	2	2	Functional hemispherectomy + hemispherotomy + Motor sparing functional hemispherectomy	Lateral/transsylvian	n/a	open	Hemimegalencephaly Rasmussen encephalitis Perinatal infarction Sturge-Weber syndrome HHE syndrome	adult + pediatric	

Abbreviations: HDP (hemispheric disconnective procedure), HHE (hemiconvulsion hemiplegia epilepsy syndrome).

## 3 History

Initial reports on the behavior of mammals after hemispheric resections were published by Goltz at the end of the 19th century (Goltz and Van Mering, 1879). Goltz described how animals interacted with the environment after he had removed large parts of the brain. One of the dogs he studied survived 18 months and was dissected. Different brain areas showed extensive atrophy and scar tissue.

In humans, it was Walter Dandy who first reported in 1928 on five patients after hemispheric resections for malignant gliomas (Dandy, 1928). All patients had a left-sided paralysis, three of them had an alteration of consciousness. Two patients died early after surgery (2 days and two weeks, respectively). Remarkably, one patient survived 3.5 years after surgery.

The first series of patients with hemispheric surgery to treat epilepsy was published in 1950 by Krynauw from South Africa (Sarat Chandra et al., 2021a; Krynauw, 1950). He reported on 12 patients, all of whom had a hemi-syndrome pre-operatively, and 10 suffered from different kinds of convulsions. The pre-surgical work-up was comprised of a surface EEG and a ventriculography in most cases. The oldest patient was 21 years old. Krynauw discussed the possible language transfer due to the early insult and the possible ipsilateral motor representation. He stated that “disorders of behavior and personality are a marked feature of this group of cases, and the profound betterment in respect of mentality in all cases exceeds our best expectations.” (Krynauw, 1950).

Rasmussen summarized the Montreal experience of hemispheric surgery in the Penfield Lecture from 1982 (Rasmussen, 1983). A high rate of early and late hydrocephalus was described as related to anatomical hemispherectomy, in some cases with a delay of ten years or more. He and his team were the first who reduced the resective proportion of the procedure and disconnected larger parts of the hemisphere, leaving the tissue in place. Seizure outcome was still promising with the less invasive procedure. He concluded that “preserving the frontal and occipital poles but disconnecting them from the rest of the brain, resulting in a functionally complete but anatomically subtotal hemispherectomy, retains the therapeutic effectiveness of a complete hemispherectomy while still protecting adequately against the serious late postoperative complication of superficial cerebral hemosiderosis and its associated neurological deterioration, hydrocephalus and sometimes death.” (Rasmussen, 1983). This was the initial spark that pushed the evolution of modern disconnecting procedures, such as lateral transsylvian or the vertical hemispherotomies (Daniel et al., 2007; Dorfer et al., 2013a).

In general, the surgical tendency to minimize exposure and complications was a move from large resections, via smaller excisions towards an almost exclusively disconnective surgical procedure without any tissue resection. The basic prerequisite for this approach was the proven effectiveness in terms of seizure outcome. This tenet does not just apply to hemispherotomy but also to more circumscribed epilepsy surgery procedures such as the posterior disconnection.

## 4 Indications

Hemispheric disconnection procedures (HDPs) are typically applied in severe or catastrophic drug-resistant epilepsies in which most or all seizures are caused by a diffusely damaged single hemisphere, after sophisticated work-up has suggested a healthy contralateral hemisphere (Schramm et al., 1995b; Villemure et al., 2000a; Lega et al., 2014a). Many patients considered for HDPs already present with hemiparesis, hemianopia and some degree of neuropsychological impairment. Severe drug-resistance is an important prerequisite for surgery.

The medical conditions for HDP can be summarized as a) acquired b) congenital and c) progressive pathologies.

### 4.1 Acquired pathologies

#### 4.1.1 Perinatal middle cerebral artery (MCA) infarction

**Fig. 1:**
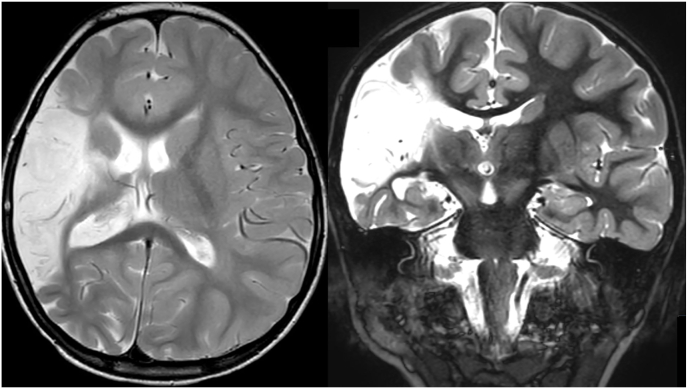
MCA-Infarct: 4yrs old male, delayed motor development, refractory seizures for 18 months; hemiparesis left, able to walk. T2 weighted MRI transversal (left) and coronar (right): Perinatal MCA-infarction involving the pyramidal tract.

Perinatal infarction (Fig. 1), or intracerebral bleeding leading to large hemispheric defects with numerous cysts, causing drug resistant-epilepsy is one of the classical indications for any type of hemispheric disconnection procedures (Kim et al., 2018). Especially in this group with stroke-induced epilepsy, an alternative disconnection procedure has been described to avoid hemispherotomy (Scavarda et al., 2009, 2010, 2018). Most patients present with a neurological deficit, and depending on the time of infarction (intra-uterine or perinatal) neurological function may already (partly) be transferred to the healthy hemisphere, rendering them ideal candidates for HDP (Marras et al., 2010). Postoperative seizure outcomes in this group are among the most promising for HDP with seizure freedom rates of more than 90 % (Schramm et al., 2012).

### 4.2 Congenital pathologies

#### 4.2.1 Malformations of cortical development (MCD)

Malformations of cortical development can be divided into two main groups: a) hemimegalencephaly (HME) and b) multi-lobar cortical malformations such as focal cortical dysplasia or polymicrogyria. Although these are two different entities, their clinical presentation and cardinal symptom is the same, namely drug resistant epilepsy (DRE). Hence, if MCDs are predominantly localized to one hemisphere, HDP can be considered as a treatment option (Lega et al., 2014b).

HME is a congenital developmental dysplastic malformation of the brain characterized by abnormal overgrowth of one hemisphere or some of its lobes, often resulting from a neuronal migration disorder. (Flores-Sarnat, 2008). The typical clinical presentation of HME includes severe DRE, contralateral motor deficit and cognitive impairment. Seizures typically begin in the early postnatal period and occur in more than 90 % of the patients. Drug resistance develops early and is typical for HME, which implies an early consideration for surgical treatment. Although hemispheric disconnection for HME shows a less seizure control compared to all other conditions treated by HDP (Di Rocco et al., 2006), patients with HME can still achieve stable long-term seizure freedom in at least 60 % of cases (Schramm et al., 2012) and improvement of cognitive abilities in selected cases (Puka et al., 2021).

### 4.3 Progressive pathologies

#### 4.3.1 Sturge-Weber-syndrome

Sturge-Weber syndrome (SWS) is a rare, sporadic neurocutaneous disease (formerly: phakomatosis) with three variants. Types 1 and 2 are easily suspected due to presence of a typical port-wine stain facial angioma, whereas type 3 only has leptomeningeal angiomatosis, and is therefore less obvious (Muralidharan et al., 2020), and presents with seizures in 75 %–90 % of the cases (Di Rocco and Tamburrini, 2006). Seizures are caused by vast pial angiomatosis, which can be localized within one lobe but often spreads over the entire hemisphere. Of note, it is presumed that the epileptogenic zone involves even larger areas of the cortex, going beyond the angiomatosis plaques (Rasmussen et al., 1972). A very informative imaging modality to delineate the extension of the (calcified) pial angiomatosis is, next to the native bone-setting computed tomography (CT)-scan and the T1W MRI with contrast, the contrast-enhanced fluid-attenuated inversion recovery (FLAIR) sequence. Approximately 60 % of the patients with SWS will become drug-resistant making them excellent candidates for disconnecting procedures if the angiomatosis plaques are strictly localized to one hemisphere. Seizure outcome is favorable as long-term seizure freedom can be achieved in 80 % of the patients (Kossoff et al., 2002).

#### 4.3.2 Rasmussen encephalitis

**Fig. 2:**
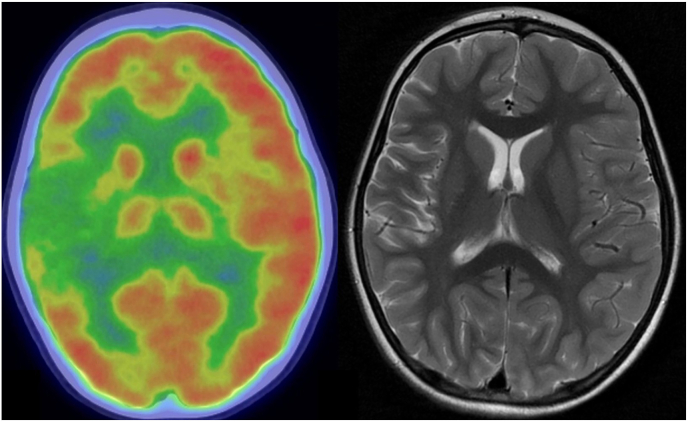
Rasmussen encephalitis: 13yrs old male, epilepsia partialis continua left arm and progressive hemiparesis. FDG-PET/CT transversal (left) and T2 weighted MRI transversal (right): Hypometabolism and slight hemiatrophy of the right hemisphere.

Rasmussen encephalitis (RE) is a very rare neurological disorder with estimated incidence rate of 2.4 cases in 1,000,000. It is characterized by inflammation of one hemisphere, progressive neurological deterioration, and cognitive decline as well as drug-resistant epilepsy. Recent findings suggest that the inflammation in RE is driven by a T-cell response (Varadkar et al., 2014). The typical course of RE includes a prodromal stage, acute stage (8–12 months) and a residual stage. The neurological deterioration and the occurrence of epilepsy typically mark the beginning of the acute stage. This stage is also accompanied by a progressive unilateral hemispheric atrophy usually starting in the insular lobe, which can be diagnosed by MRI (Fig. 2). RE usually affects only one hemisphere, despite its patho-immunological background. Progressive neurological deterioration ending with hemiplegia (as well as aphasia in the dominant hemisphere) and epilepsy are cardinal symptoms of the disease. Drug-resistance occurs in almost all cases, and about 50 % of the patients with RE will develop epilepsia partialis continua (EPC). HDP represents the only cure for seizures. Despite the unilateral nature of the disease and the early development of DRE, the decision and especially the timing of surgery is difficult since most patients initially present with only mild hemiparesis. Furthermore, immunosuppressive or immunomodulatory treatment has been shown to slow the disease and can be considered as suitable treatment prior to surgery especially in patients with slow disease progression and mild or no neurological symptoms (Bien and Schramm, 2009; Bien et al., 2013). These treatment regimens seem to slow the disease but they are unable to cure RE and it is a matter of debate, whether they can improve long-term outcome. Even more importantly, immunosuppressive, or immunomodulatory treatments have little to no effect on epilepsy. Therefore, immune treatment modalities should not be a reason to postpone surgery, especially if RE develops rapidly. Thus, a functional HDP remains the only possible cure for seizures caused by RE, a decision, which must be weighed against the neurological impairment including hemiplegia and hemianopia (Bien and Schramm, 2009). Of note, most of the patients achieve independent walking after rehabilitation, while fine movements of the fingers remains impaired, as generally occurs in HDP for other indications. The decision and timing of surgery on the language dominant side is even more challenging and therefore it is advisable, depending on the age of the patient, to proceed with a thorough investigation with fMRI or Wada tests to be able to estimate the risk for postoperative language impairment. Recent publications demonstrated that, although in pre- and young adolescent patients with RE, a functional HDP on the left (dominant) side, causes language function worsening in the acute postoperative phase, over the long-term these language functions can recover under intensive rehabilitation care (Bulteau et al., 2015, 2017; Ivanova et al., 2017).

## 5 Timing

The optimal timing of the procedures depends mostly on the severity of seizures and the underlying pathology. In young infants with very severe epilepsy caused by acquired pathologies, or malformations of cortical development, who present with already impaired neurological function, and who have a high incidence of sudden unexpected death in epilepsy (SUDEP), decision-making can be straight-forward. In very young children surgery can be performed in experienced epilepsy surgery centers despite the fact of low weight and blood volume, as reported by Roth et al. (2021). Other experienced centers reported that HDP can be performed in infants from 4 months onward (Schramm et al., 2012) and may push the limit to 2.5 months (Dorfer et al., 2015). The earlier the surgery, the better the long-term outcome, since hemispherotomy represents a highly successful treatment option for seizure outcome, and seizures cessation will prevent the development of epileptogenic encephalopathy, which could negatively affect the healthy hemisphere as well (Althausen et al., 2013). A recent multicenter and multinational study evaluated ultra-early epilepsy surgery before the age of 3 months. The study included 48 hemispheric surgeries. They found that ultra-early surgery was not associated with more permanent morbidity or mortality than surgery in older infants (Roth et al., 2021).

The timing of surgery in progressive pathologies like SWS and especially in RE is more challenging. Here, one should weigh the instant loss of neurological function due to the disconnecting procedure against the seizure burden and the risk for development of epileptogenic encephalopathy. This decision should be made on an individual basis. However, if the seizures are incapacitating one might be more prone to go for earlier surgery to at least preserve the possibility for transfer of neurological function to the healthy hemisphere before it has been damaged by ongoing seizures (Bien and Schramm, 2009; Guan et al., 2017).

Timing is also a challenge in children older than 5–7 years with underlying pathological conditions of the dominant hemisphere. Recent literature shows that language lateralization by resting state-fMRI is possible in children and can be helpful in the preoperative counseling (Pur et al., 2021). In general, experience shows that hemispheric disconnecting procedures are safe in terms of cognition at different ages regardless of the language dominance and that the postoperative outcome often resembles the preoperative condition (Althausen et al., 2013; Silva et al., 2020).

### 5.1 Techniques

Since the first description in 1928 of an anatomic hemispherectomy for a right hemispherical tumor by Walter Dandy (1928), the first description of this surgical procedure for epilepsy by McKenzie in 1938 (McKenzie, 1938) and the publication of Krynauw in 1950 of a pediatric patient cohort after hemispherectomy (Krynauw, 1950), major changes in the technique have occurred, reflecting technological advancements and physiological – anatomical understanding. After several decades with a scarcity of publications, a new era started in the mid-1990s with several publications on the diversity of hemispherectomy and hemispherotomy techniques, and their respective outcomes and complications.

#### 5.1.1 Anatomical hemispherectomy

In the early days, this surgical procedure consisted of removal of an entire cerebral hemisphere sparing the basal ganglia for oncological (Dandy, 1928) or epileptological (Krynauw, 1950; McKenzie, 1938) indications. Theodore Rasmussen (1910–2002), successor of Wilder Penfield as head of the Montreal Neurological Institute, and his group described the “en bloc” technique of resecting an entire hemisphere (French, 1955/Rasmussen, 1983). The previous technique was the piecemeal removal of the different lobes, as described by Dandy, Penfield and Krynauw respectively (Dandy, 1928; Krynauw, 1950; Penfield et al., 1979).

Due to the necessarily large cerebral exposure, the wound and craniotomy are accordingly large. The craniotomy is planned in an anteroposterior (AP)-direction from the frontal towards the occipital pole and in a craniocaudal direction from almost the vertex towards the base of the middle cranial fossa. After this extensive hemicraniotomy, the dura is opened in different flap-directions almost up to the superior sagittal sinus. The middle and anterior cerebral arteries are divided and clipped, sparing the deep perforators of both as well as the large parasagittal bridging veins, since not all of them should be closed in the beginning because of the risk of brain swelling. The hemisphere can subsequently be retracted to visualize the corpus callosum. The callosotomy is performed, after which the frontal horn is entered. From the most anterior part of the ventricle a frontobasal disconnection is performed. Thereafter the ventricle is followed to the trigone, temporal horn and anterior hippocampus. Most frequently the hemispherectomy is performed in more than one “en bloc” step in which, after isolating the basal ganglia block, the frontal and temporal lobes are removed, followed by the parieto-occipital lobes. In most procedures the insular cortex is also removed (Schramm, 2002). This “classical” anatomical hemispherectomy is nowadays still performed in a very minority of cases in some epilepsy surgery centers, especially in cases of recurring seizures or secondary to HME with a significantly distorted anatomy (Di Rocco and Iannelli, 2000; Sood et al., 2019).

Despite its effectiveness for seizure outcome, this technique is very time-consuming and appears to be associated with several complications, specified below.

In 1968 and 1983 some anatomically “less-radical” modifications were described by authors such as Ignelzi et al. (Ignelzi and Bucy, 1968) and Adams et al., who respectively proposed the so-called hemidecortication in which “only” the epileptogenic cerebral cortex is removed (Ignelzi and Bucy, 1968), and the reduction of the volume of the resection cavity by plugging the foramen of Monro and suturing the convexity dura to the falx and tentorium, which reduced the rate of hydrocephalus complications (Adams et al., 1988; Adams, 1983).

#### 5.1.2 Functional hemispherectomy

These less invasive options had also several disadvantages which led most epilepsy surgery centers, from the mid-1990's, to adopt the non-anatomical, i.e., the functional hemispherectomy or hemispherotomy procedure. This procedure, first performed by Rasmussen in 1974, was an improvement to the anatomical hemispherectomy (Rasmussen, 1973), and since then many surgical nuances have been described and will be explained below in a chronological order. All these less-invasive, less resective and more disconnective procedures have in common that, tracking/tracing along unilateral efferent and commissural anatomical structures, the following are progressively disconnected: corona radiata and internal capsule, frontobasal, and temporomesial structures as well as the insula and corpus callosum (Young et al., 2020). To obtain seizure freedom it is critical to interrupt different unilateral, efferent, projective fiber tracts, e.g., the cortico-spinal tract, but also commissural fibers i.e., the corpus callosum or the hippocampal commissure have to be interrupted in order to prevent seizure spread to the other hemisphere. These more disconnective, less resective procedures are done to lessen complications, especially the development of hydrocephalus.

#### 5.1.3 *Rasmussen's functional hemispherectomy* (Rasmussen, 1973)

The key principles of the Rasmussen approach were the resection of a large area between fronto-dorsal (F1/F2/F3) and anterior parts of the superior and inferior parietal lobules, a temporal lobectomy, a callosotomy and disconnection of the rest of the frontal/parietal and occipital lobes (Rasmussen, 1973). After Rasmussen's description four other functional hemispherotomy techniques were described.

1. *Vertical parasagittal hemispherotomy* (Delalande et al., 1992, 2007; Danielpour et al., 2001; Giordano et al., 2015; Baumgartner et al., 2017)

**Fig. 3:**
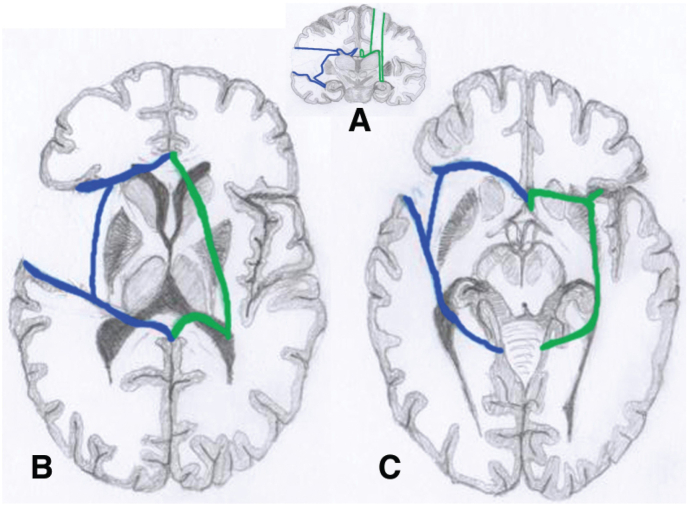
Schematic drawings of the 2 hemispherotomy techniques. Each hemisphere shows a single technique with the left hemisphere *(blue)* representing the periinsular hemispherotomy and the right *(green)* representing the vertical parasagittal hemispherotomy.A: Coronal view. B and C: Axial views. (In order to have only 2 axial cuts, some structures are represented on the same schema even if they are anatomically at different levels).(with CR permission O.Delalande and JNS group).

**Fig. 4:**
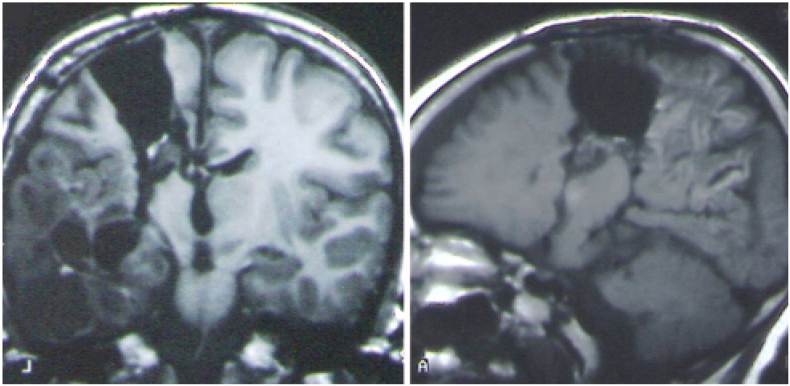
Coronal *(left)* and sagittal *(right)* T1-weighted MR images of a vertical parasagittal hemispherotomy. (with CR permission O.Delalande and JNS group).

This technique was first described by Delalande et al., in 1992. The patient is in a supine position, with the head fixated orthograde, in slight anteflexion. Neuronavigation is recommendable, especially in cases with a distorted anatomy, to perform a predominantly precentral localized craniotomy in order to have sufficient access for the anterior disconnection. With an average length of 5 cm and width of 3 cm, a frontal cortex resection is performed and through dissection of the white matter the lateral ventricle is entered. At the level of the roof of the lateral ventricle, the corpus callosum is encountered and the callosotomy is started with the corpus and splenium and completed with the genu and rostrum till the level of the anterior commissure. At the level of the trigone, the posterior fornix is cut to disconnect the hippocampus. The vertical disconnection line is performed lateral to the basal ganglia and thalamus, following the choroid plexus to the anterior part of the temporal horn where the amygdala is resected. The fronto-(basal) disconnection is performed by resection of the posterior gyrus rectus and frontobasal white matter until the anterior cerebral artery (ACA) and optic nerve can be visualized, after which an incision is performed to disrupt the fibers between anterior temporal horn and frontobasal cortex (Delalande and Dorfmüller, 2008). Further technical variants have been proposed by Danielpour (Danielpour et al., 2001), Giordano (Giordano et al., 2015) and Baumgartner (Baumgartner et al., 2017) aiming to avoid or at least reduce the access to the ventricular system. In the vertical midline approach, a coronal incision of <10 cm is made above the coronal suture, followed by a 6 cm wide craniotomy that exposes the superior sagittal sinus. The first step is an interhemispheric approach allowing complete corpus callosum exposure. Callosotomy is then performed from the rostrum with the visualization of the pericallosal artery, to the splenium with visualization of the internal cerebral veins/vein of Galen confluence. Extraventricular dissection in the reflection line of the septum pellucidum avoids cerebrospinal fluid (CSF) leakage, and brain collapse at this time. Then the ventricle is opened, and lateral disconnection is performed from the temporal horn (behind the plane of the glomus of the choroid plexus) to the frontal horn, lateral to the choroid plexus that delineates the lateral limit of the thalamus, in a posterior to anterior direction until the anterior choroidal point. Anterior disconnection is then performed starting on the midline from the genual part of the cingulum, subpially following the ACA to the frontal base, and then moving laterally to the carotid bifurcation. This disconnection goes back to the level of the anterior commissure, which is a major difference to the lateral technique (Fallah et al., 2021). The temporal disconnection is reached while following the vessels until the anterior choroidal point. The fimbria is disconnected posterolaterally to the splenium of the corpus callosum, in the medial wall of the ventricle atrium. At the end of the disconnection, the ventricular access is closed with fibrin glue, following irrigation of the ventricular cavities with saline after meticulous hemostasis, to avoid subdural collection (Fig. 3 and 4).

2. *Periinsular hemispherotomy* (Villemure and Mascott, 1995; Villemure et al., 2000b)

The patient is placed in a supine position with the head turned almost horizontally. This technique uses the transventricular approach for the mesial hemispheric disconnection in the same manner as the keyhole transsylvian approach (Fig. 3)(see below). The craniotomy is centered over the complete Sylvian fissure, exposing the suprasylvian circular sulcus as well as the frontal and temporal operculum. As the name says, two peri-insular windows for access to the frontal and the temporal ventricles are performed by resection of frontal and temporal operculum (T1) respectively. In the suprainsular window it is necessary to open the frontal horn in the entire AP direction (frontal horn - trigone) by resection of the fronto-parietal opercular cortex and thereby also transecting the corona radiata. From inside the ventricle, a complete callosotomy is performed with a disconnection at the splenial level of the fornix-fimbria hippocampi connection (psalterium-commissura hippocampalis) and an anterior frontal lobe disconnection from the rostrum in the direction of the sphenoid wing. In the infrainsular window, the superior temporal gyrus (T1) is resected from the uncus to the posterior part of the insula. Via the inferior circular sulcus, the temporal horn is entered, and a resection of uncus, lateral amygdala and anterior hippocampus is performed. Finally, the insular cortex is aspirated and the insula is disconnected at the level of the claustrum/external capsule.

An alternative way to perform the peri-insular hemispherotomy is by entering the “C” shaped ventricle by a “C” shape corticectomy through F3, supramarginal gyrus, and T2 – then disconnecting the tissue from F3 to the Sylvian fissure at the level of the foramen Monro, and from T2 to the Sylvian fissure at the most anterior point of the temporal horn. At this stage, the insula is undermined at the level of the external or extreme capsule, the MCA is coagulated and transected at the level of the insular vallecula, and the entire peri-insular and insula are removed en bloc.

3. *Transsylvian keyhole functional hemispherectomy* (Schramm et al., 1995a, 2001)

This is the third described technique and a surgical variation on the previous one. The technique was described in detail by Schramm in 1995 (Schramm et al., 1995a), being supplemented by patient details and surgical outcome in 20 cases (Schramm et al., 2001).

The most essential surgical steps in this keyhole procedure are the following: 1. a curvilinear frontotemporal incision and a relatively small craniotomy centered over the entire Sylvian fissure and insula exposing the Sylvian fissure at the inferior part of the craniotomy. This technique is therefore especially suitable for cases with a certain amount of brain atrophy, such as patients with perinatal ischemic events, and RE cases. Craniotomy size (varying from 4 × 4 cm to 5 × 6 cm), preferably guided by neuronavigation, depends, among other things on the AP length of both corpus callosum (mostly 6.5 cm), insula (limen insulae)-basal ganglia block (pulvinar thalami) and the size of the ventricular system. 2. Then the Sylvian fissure is opened and both the inferior and superior circular sulci are visualized. Anatomically the frontal operculum can cover the superior circular sulcus for more than 3 cm whereas this is less so for the temporal operculum and the inferior sulcus (0.5–1.0 cm). The temporal horn is opened via the limen insulae (inferior sulcus) and an unco-amygala-hippocampectomy is performed. Subsequently the temporal horn is further opened following the circular sulcus to the frontal horn. 3. Via the tip of the frontal horn, a frontobasal disconnection is performed between the arachnoid covering the landmarks ACA basally in the midline and the MCA laterally. 4. Now, via the same route back from the frontal horn in the direction of the trigone, a complete intraventricular callosotomy is performed until the splenium is reached. To guide the callosotomy, in the first part, more anterior, the ACA branches are followed. When these arteries become too thin to follow, the falx can be used as a guide. In the splenial region, the falco-tentorial junction is followed down through the hippocampal tail to the temporal horn where the procedure started. All major arterial branches of MCA, ACA and posterior cerebral artery (PCA) are spared. Finally, the insular cortex is aspirated. One indication for not performing the keyhole procedure would be HME because of the enlarged hemisphere and atypical insular cistern. In these cases, the best solution is a temporal lobectomy or a frontoparietal operculum resection. With these resections, the surgery will be faster and the occurrence of brain swelling less problematic. Care should be taken to strictly avoid injury to the contralateral healthy hemisphere.

4. *Japanese modified periinsular hemispherotomy* (Shimizu and Maehara, 2000)

This surgical technique combines different aspects of the peri-insular technique and parts of the technique described by Delalande. The craniotomy is centered over the entire AP direction of the lateral ventricle. Resection of frontal operculum and corticectomy of the insular upper half is carried out. Via the white matter, a route is created to the lateral ventricle and from inside a complete callosotomy is performed. The procedure ends with a temporo-mesial disconnection by an amygdalo-hippocampectomy.

All these surgical disconnection techniques can be applied in patients with HME but specific attention should be paid to the more voluminous diseased hemisphere which sometimes preoperatively shows signs of midline displacement, displacement of the superior sagittal sinus to the opposite side and a much more complex anatomy because of dysplastic brain tissue, leading to a less demarcated gray-white matter transition zone. Therefore, in general, the recommendation is to perform a larger craniotomy and volume of tissue resection, e.g., a standard temporal lobectomy. Sometimes in a larger anatomical resection, the MCA or ACA can be clipped, which may decrease blood loss (Sood et al., 2019), but could increase postoperative ischemia and brain edema.

#### 5.1.4 Postoperative management

All patients should be transferred to a dedicated special care ward (medium or intensive care) for at least one night. Pediatric patients, and especially young infants, may need a blood transfusion. Almost all patients have a (slight) rise in body temperature, explained by “aseptic meningitis” and caused by CSF contamination with blood products. Routine external ventricular drainage (EVD) has been shown to reduce the rate of postoperative fever and hydrocephalus (Lu et al., 2023a). In some centers a drain is left in situ for a couple of days till the CSF clears, but there is no recommendation based on clear evidence. Intraoperative extensive rinsing before closing might contribute to avoid postoperative hydrocephalus.

### 5.2 Techniques – new developments

In the last 8 years some reports have described variations of the hemispherotomy technique. Kawai et al. described a variation for the vertical hemispherotomy, applied in 7 patients, with the main difference to Delalande's technique being that the authors used the interhemispheric, instead of transcortical, route. The second modification was the target for the anterior dissection plane, namely the anterior end of the foramen of Monro instead of the subcallosal area. They reported a good seizure outcome (International League Against Epilepsy (ILAE) Class 1 in 6 out of 7 patients) and less brain resection (Kawai et al., 2014; Uda et al., 2016). An alternative variant for the hemispheric disconnection via an extraventricular route was described by Giordano et al. (2015). These authors developed this alternative procedure especially to reduce the chance of postoperative hydrocephalus.

#### 5.2.1 *Endoscope-assisted hemispherotomy* (Wagner et al., 2018)

In 2018 Wagner et al. (2018) reported on an endoscope-assisted functional hemispherotomy in two pediatric cases with chronic epilepsy due to perinatal strokes. Other reports on endoscope-assisted disconnective surgery have been published since (Baumgartner et al., 2017; Bahuleyan et al., 2010; Sarat Chandra et al., 2015; Chandra et al., 2016; Sood et al., 2015). The main advantage is the smaller incision and craniotomy. Essentially, the surgery is identical to the vertical parasagittal approach. A linear paramedian incision, 2 cm anterior and 3cm posterior to the coronal suture is made, followed by a 4 cm long and 2 cm wide craniotomy. Following an interhemispheric complete callosotomy, the lateral ventricle is entered, and an anterior or frontobasal disconnection followed by the middle and posterior white matter disconnection between frontal and temporal horns up to the trigone. Finally, a hippocampal disconnection is performed (resecting the amygdala and disconnecting the posterior fornix). Due to long surgery duration with this procedure, a higher complication rate, like e.g., infections, could perhaps be associated compared to open surgery, but is in the scarce literature not clearly described.

#### 5.2.2 *MRgLITT and radiofrequency ablation for functional hemispherotomy* (Chua et al., 2020)

MRI guided laser interstitial thermal therapy (MRgLITT) enables real-time image-guided ablation of the predefined brain tissue region by applying laser energy via one or more stereotactically inserted laser probe(s). In 2012 Curry et al. (2012) first reported LITT therapy in 5 pediatric patients with different lesions. In the following years, different epileptogenic lesions like periventricular heterotopias (Esquenazi et al., 2014), focal cortical dysplasia, tuberous sclerosis (Lewis) and hypothalamic hamartomas have been treated by LITT, but there are still very few studies report on (long-term) outcome. A recent systematic review on corpus callosotomy by this technique was published with the conclusion that all included studies on this topic only reach class IV evidence, so prospective trials are necessary to compare its effectiveness with that of standard open callosotomy (Badger et al., 2020). This, relatively new, “minimally invasive” treatment method with curative potential for chronic, drug-resistant, epilepsy was recently performed and described in a pediatric case whose multiple comorbidities consequently made it not suitable for open disconnective surgery. Crucial brain areas to be disconnected were established and disconnection could be performed by implantation of 5 laser catheters throughout the hemisphere. As this was the first case, no definitive conclusion or advise can be given at present (Chua et al., 2020). More recently, Chandra has applied a robotic thermocoagulation technique using radiofrequency (RF) ablation with the same technical concept of MRIgLITT (Sarat Chandra et al., 2021b).

## 6 Complications: an overview and a consensus on their avoidance

Thorough knowledge of any procedure-associated complication cannot be overemphasized – this guides the surgeon to making an accurate and mature decision regarding surgery for the candidate, while allowing for early detection and proper management of any problems. Moreover, understanding of the underlying pathophysiological mechanism for each complication provides the opportunity for developing avoidance or mitigation strategies, improving the safety profile and overall outcome of a surgical procedure.

Anatomical hemispherectomy constitutes the only therapeutic epilepsy surgery procedure that has been associated with mortality. Previous publications have reported mortality rates varying between 2 and 10 % (Fountas et al., 2006). The extensive nature of hemispherectomy, the removal of multiple lobes, massive intraoperative blood loss, mainly due to sagittal sinus lacerations, and the sacrifice of multiple cortical veins leading to extensive, acute postoperative edema were a few of the causes of mortality. However, more recent clinical series have demonstrated that the mortality associated with various hemispherotomy techniques is minimal. Schramm et al. reported a mortality rate of 1.0 % in a large retrospective pediatric series while in the adult series of the same center the reported mortality rate was 0 % (Schramm et al., 2012; Althausen et al., 2013). Likewise, several recent pediatric series have reported 0 % mortality (Dorfer et al., 2013b; Moosa et al., 2013; Ye et al., 2020). The transformation of the procedure from an extensive resection to a minimal disconnection, the avoidance of cortical vessel coagulation of the pathological hemisphere, and the advances in neuroanesthesia during the last two decades may well explain the minimization of the associated mortality.

Multifactorial morbidity has also been associated with the various hemispherectomy techniques, compromising their safety profile and limiting clinical use. The reported complications could be grouped into surgical, neurological, neuro-endocrinological and neurocognitive, mostly for analytic purposes. Superficial cerebral hemosiderosis, significant intraoperative blood loss requiring massive blood transfusions, the development of postoperative hydrocephalus necessitating shunt insertion, postoperative hematoma formation, persistent postoperative fever, and postoperative infection represent the most common reported surgical complications, especially following anatomical hemispherectomy. The existence of superficial cerebral hemosiderosis, a commonly reported complication in the original series, has been questioned by many authors (Schramm et al., 2012; Di Rocco and Iannelli, 2000). Indeed, all recently published series, either pediatric or adult, have reported no such cases, making the discussion about cerebral hemosiderosis of historical value only (Schramm et al., 2012; Althausen et al., 2013; Sood et al., 2019; Fountas et al., 2006; Di Rocco and Iannelli, 2002), probably due to the minimization of tissue destruction and blood loss. As a neuro-endocrinological complication due to hypothalamic injury during the frontal disconnection, SIADH or DI could occur and lead to severe life-threatening hypo- or hypernatremia and even to sinus thrombosis (Saito et al., 2020a).

A major concern regarding hemispherectomy has undoubtedly been the massive intraoperative blood loss and its serious metabolic sequences (induced tachycardia, circulatory instability, metabolic acidosis, hypothermia, severe electrolytic abnormalities etc.). The severity of intraoperative blood loss is reflected in the necessity for intraoperative blood transfusion. Brian et al. (1990) reported in an older series that all of their cases required blood transfusion. Similarly, Gowda et al. (2010), in a more recent series, reported that blood transfusion was necessary in all their cases. It has to be pointed out, however, that their series included solely pediatric patients aged under 6 months. Similarly, Roth et al. reported on children undergoing hemispheric surgeries before the age of 3 months, and all received blood transfusions (Roth et al., 2021). Since the era of hemispherotomy (minimization of brain tissue resection) instead of hemispherectomy (partial or complete hemispheric brain tissue resection) reported blood loss and the necessity for blood transfusion has decreased, especially with the lateral keyhole approaches (Schramm et al., 2001) and in the group operated via the vertical technique (Dorfer et al., 2013b), probably because of smaller skin incisions and brain exposure.

The incidence of postoperative hydrocephalus comprises another worrisome complication associated with hemispherotomy. Lee et al. (2014) in their review article reported a 9–81 % hydrocephalus incidence in mixed populations (adult and pediatric), while the respective percentage in pediatric series was 23 %. Similarly, Brotis et al. (2019) reported 2–26 % postoperative hydrocephalus in their systematic review. Recently published pediatric series reported lower hydrocephalus incidence around 13–14 % (Weil et al., 2020), while Lopez et al., in their review study found an overall 19 % hydrocephalus incidence (Lopez et al., 2021). It has been postulated that certain pathological entities such as HME and multifocal cortical dysplasia are more frequently associated with the development of postoperative hydrocephalus, and the subsequent need for shunt insertion. The insertion of an EVD does not seem to reduce the incidence of definitive post-operative hydrocephalus (Sood et al., 2019), though other centers show a reduction of post-operative hydrocephalus (Lu et al., 2023b).

The formation of a postoperative hematoma, infection (either a localized surgical wound infection or meningitis), and/or the occurrence of postoperative fever are frequently reported as cumulative surgical morbidity among many series, both in cases with hemispherotomy as well as hemispherectomy. Schramm et al. reported rates of 7.4 % in their adult series, and somewhat higher, 9.7 %, in their pediatric series (Schramm et al., 2012; Althausen et al., 2013). Likewise, Ye et al. (2020) reported hematoma/hygroma/infection rates of 10 %, while in the Weil et al. (2020) pediatric series the percentage was 14.8 %. Interestingly, Santos et al. reported 28.5 % surgical complications in their series. It has to be pointed out that they only included reoperations (Silva et al., 2020). Lopez et al., in their review study including 37 pediatric series, found that the hematoma formation rate varied between 10 and 36 %, and the incidence of postoperative infection ranged from 2 % to 7 %, while persistent postoperative fever was observed in up to 83 % of cases (Lopez et al., 2021). The authors also found that certain pathological entities such as HME and SWS are more frequently associated with hematoma formation, while RE may predispose to the occurrence of postoperative fever (Lopez et al., 2021). Kamath et al. have reported increased incidence of post-operative fever in patients with RE, while patients with underlying pathologies such as cortical dysplasia or polymicrogyria tended to have less severe fevers (Kamath et al., 2015). The authors also found that the usage of an EVD may mitigate the possibility of post-operative fever (Kamath et al., 2015). DiRocco et al. postulated that younger age may predispose to increased surgical complications (Di Rocco and Iannelli, 2000; Fountas et al., 2006).

Despite the extent of disconnection and resection of cerebral tissue in hemispherotomy the incidence of unexpected neurological complications respectively sequelae are quite low. Permanent worsening of a pre-existing hemiparesis or de novo development of hemiparesis has been reported in the range of 8–21 % (Gowda et al., 2010; Ramantani et al., 2013; Ghatan et al., 2014; Schusse et al., 2018). More specifically, Gowda et al. (2010) reported a worsening of hemiparesis in 8 % of their pediatric cases, Ramantani et al. (2013) found such worsening in 10 %, as well as Ghatan et al. (2014). This percentage was higher in the adult series of Schusse et al. (2018), who reported a 21 % incidence. It has to be mentioned however, that even in cases with worsening of the preoperative hemiparesis, patients remained ambulatory after surgery. Postoperative worsening of language/speech was observed in 10 % of the adult cases (Schusse et al., 2018). There are reports of temporary mutism, which spontaneously resolved (Schusse et al., 2018). These symptoms occurred more frequently in dominant hemisphere involvement. Postoperative visual field and/or visual acuity worsening has been demonstrated in pediatric series (Chen et al., 2019; Meer et al., 2021). Chen et al. (2019) reported that 49 % of their pediatric cases developed de novo or had worsening of their preoperative strabismus. It is of interest, that the vast majority of these patients developed torticollis from compensating for their visual deficits (Chen et al., 2019). Similarly, Meer et al. (2021) reported in their pediatric series that 56 % of their cases had decreased visual acuity after surgery, 71 % experienced new visual field deficits while visual field impairment preexisted in others (Meer et al., 2021). In summary, a postoperative hemianopia is unavoidable in a complete hemispheric disconnection, yet in most cases it will not impair function, and various compensatory mechanisms have been described, so correction (such as treatment of the strabismus) may not be necessary. The degree of speech/language deterioration as well as loss of motor function on the contralateral side is much more difficult to predict in the preoperative counseling with the patient and family; however, especially in younger children, linguistic improvement is expected, even beyond the preoperative status.

The effect of hemispherotomy on neurocognitive status has not been adequately explored. The absence of such reports cannot however be considered as lack of such an impact. This issue remains to be more accurately studied in the future.

The role of the type of hemispherotomy technique in the development of certain complications is of great interest. The comparison between the existent series is extremely difficult due to different patient populations, underlying pathology, age of seizure onset, impact of various anti-seizure medication (ASM), and the utilization of various surgical techniques even in the same clinical series (Limbrick et al., 2009; Kwan et al., 2010; Iwasaki et al., 2015). Limbrick et al. (2009) in their pediatric series found no difference in the incidence of complications between the various surgical hemispherotomy techniques employed. On the other hand, Kwan et al. (2010) reported that the peri-insular technique was associated with fewer complications. On the contrary Iwasaki et al. concluded in their pediatric series that the vertical hemispherotomy technique was safer, since it was associated with less frequent perioperative complications (Iwasaki et al., 2015). It is apparent that the extraction of any statistically powerful conclusions from these series would not be very meaningful, given the limited number of participants, retrospective nature, and non-homogeneous character of these studies.

One important complication eventually leading to death could be an electrolyte imbalance, mainly caused by syndrome of inappropriate secretion of anti-diuretic hormone (SIADH), cerebral salt wasting, or diabetes insipidus due to hypothalamic ischemia when coagulating perforating arteries of the anterior communicating artery complex and the ACA when performing the frontobasal disconnection. Severe hypo- or hypernatremia and refractory brain edema may result in life threatening comatose state and death (Saito et al., 2020b).

## 7 Current practice in Europe

In the context of this manuscript, a survey with 12 to-the-point questions regarding the current practice of hemispheric disconnection procedures was composed and sent to different European centers performing epilepsy surgery. These centers were geographically well spread over Europe. The detailed survey answers are demonstrated in Table 1 and summarized below.

In most of the countries participating in this study, the practice of epilepsy surgery is centralized and, in most centers (N = 20/27, 74 %), only one neurosurgeon performs this type of complex surgery. Most centers started this surgery between 1980 and 2000 (N = 16/27 centers; 59 %), eight centers (37 %) between 2000 and 2010 and three centers (11 %) began performing this surgery after 2010. The two major techniques performed in all centers are the lateral transsylvian or the vertical parasagittal method with associated variants. Which of either technique is performed strongly depends on the center in which the epilepsy surgeon was trained. In 13 centers (n = 13/27; 48 %) only the lateral transsylvian technique was used, in eight centers (n = 8/27; 30 %) only the vertical parasagittal technique and six centers (22 %) used both techniques. Thirteen centers (48 %) treated only pediatric patients, thirteen centers (48 %) treated both pediatric and adult patients and one center (4 %) only adult patients. The “classical” causes for (catastrophic) drug-resistant epilepsy from one hemisphere were seen and treated in almost all centers (96 %). Finally, regarding the frequency of these complex surgeries per year, 15 centers (56 %) reported an increase, nine centers (33 %) no change and three centers (11 %) a decrease in these surgeries over the last 5–10 years.

We would like to emphasize that hemispheric surgery is an extremely complex intervention. It requires a highly experienced interdisciplinary team and specialized intraoperative and postoperative facilities to guarantee a favorable surgical outcome. Before performing hemispheric surgery, the epilepsy surgeon should undergo an elaborate exhaustive training by a colleague with profound experience in this sort of interventions/procedures. With hemispheric surgery, training in collaboration with other specialized centers is common practice.

## 8 Strengths and limitations

This manuscript is the result of an international European collaborative effort reflecting on hemispheric disconnective procedures in drug-resistant epilepsy supported, on the one hand by peer-reviewed published literature, and on the other hand, the experience accumulated by the authors over the past years. Though the available literature is vast, it is heterogenous and for this review used to supplement and substantiate statements. Given the complex but rare pathology encountered in hemispheric disconnective procedures, a high level of evidence is very difficult to achieve. Therefore, we asked neurosurgeons from renowned European epilepsy surgery centers to shed light on the various topics discussed and to provide a summary for current practices, under the authority of the EANS functional neurosurgery section.

## 9 Conclusion

This is the first paper to report a European consensus statement regarding the history, indications, techniques and complications of hemispheric disconnective procedures for different causes of chronic, drug-resistant epilepsy. Furthermore, a unique overview of the current practice of these surgical procedures in renowned, European epilepsy surgery centers is presented here. This overview provides the insight that, currently, only open surgical disconnective procedures, with different technical variations, can deliver a long-term postsurgical seizure outcome. Although minimally invasive surgical techniques in the field of epilepsy are rapidly developing and reported in single case reports or small case series, the long-term seizure outcome is not yet known and will hopefully be reported in the near future through collaboration of high-volume epilepsy surgery centers.

## Funding, grant/award

No funding was received for this research.

## Code availability

Not applicable.

## Ethical approval statement

Not applicable, this manuscript does not contain any research studies with human participants by any of the authors.

## Consent for publication

Not applicable.

## Author contributions

This manuscript was initiated by OEMGS, who had the original idea. Literature search and application of the Preferred Reporting Items for Systematic Reviews and Meta-Analyses (PRISMA) guidelines was performed by RHGJvL and OEMGS. The writing of the paragraphs on the different topics was performed by OEMGS, MvL, DD and KF, all members of the EANS Functional Neurosurgery section. OEMGS contacted the other co-authors and sent around the questionnaire on the current practice in Europe. All the co-authors critically revised the manuscript and gave a substantial contribution in the improvement of the content of this manuscript.

## EANS section functional neurosurgery members/collaborators

Antonio Goncalves Ferreira, Department of Neurosurgery, Hospital Santa Maria, Lisbon, Portugal.

Linda Ackermans, Department of Neurosurgery, Maastricht University Medical Center, Maastricht, The Netherlands.

Niels van der Gaag, Department of Neurosurgery, Haaglanden medical center, The Hague, The Netherlands.

Tipu Aziz, Department of Neurosurgery, John Radcliffe hospital, Oxford, UK.

Francois Alesch, Department of Neuromodulation, Zwickau, Germany.

Yaroslav Parpaley, Department of Neurosurgery, University hospital Bochum, Germany.

Mike Hart, Department of Neurosurgery, St George's university hospitals, London, UK.

Ersoy Kocabicak, Department of Neurosurgery, University Samsun, Samsun, Turkey.

Pedro Duarte Batista, Department of Neurosurgery, Hospital da Luz, Lisbon, Portugal.

Josue Avecillas-Chasin, Department of Neurosurgery, University of Nebraska Medical Center, Omaha, Nebraska, USA.

Andrey Sitnikov, Department of Neurosurgery, Federal centre of treatment and rehabilitation, Moscow, Russia.

Oystein Tveiten, Department of Neurosurgery, Haukeland university hospital, Bergen, Norway.

Ido Strauss, Department of Neurosurgery, Tel Aviv Sourasky Medical Center, Tel Aviv, Israel.

## Declaration of competing interest

The authors declare that they have no known competing financial interests or personal relationships that could have appeared to influence the work reported in this paper.
